# The Mobile Alzheimer's Disease Research Evaluation (MADRE) Model to Engage Understudied Communities

**DOI:** 10.1002/alz70858_101153

**Published:** 2025-12-25

**Authors:** Rosie E Curiel Cid

**Affiliations:** ^1^ University of Miami Leonard M. Miller School of Medicine, Miami, FL, USA

## Abstract

**Background:**

The impact of Alzheimer's Disease (AD) and Alzheimer's Disease‐Related Dementias (ADRD) is projected to rise significantly among Hispanic/Latino (H/L) and Black/African American (B/AA) populations. However, these groups remain underrepresented in clinical research and trials, resulting in critical gaps in understanding the effectiveness of early‐stage detection and monitoring methods for AD/ADRD. The lack of representation in studies focused on the earliest stages of these diseases limits the ability to assess the diagnostic, prognostic, and clinical utility of promising biomarkers and their effects on cognition, making it difficult to establish generalizable findings.

**Method:**

The MADRE (Mobile Alzheimer's Disease Research and Evaluation) study was designed to provide comprehensive evaluations in participants' homes or community centers, specifically targeting B/AA and H/L populations. This approach reduces participant burden, increases the standardization of evaluations, and ensures efficient and accurate data capture. By meeting participants in familiar settings, the study minimizes the stress of traveling to medical centers, improving accessibility for individuals in underserved regions who may not be aware of research opportunities.

**Results:**

In an ongoing longitudinal study aimed at deep‐phenotyping B/AA older adults, we successfully recruited 159 participants over 18 months. The cohort consisted of 72 males and 84 females, with an average age of 67.32 years (SD = 5.5; range = 52–88). The annual retention rate exceeded 93%. The sample included 33% with mild cognitive impairment (MCI), 30% with mild impairments but not meeting MCI criteria (“impaired, not MCI”), 3% with subjective cognitive complaints, and 34% cognitively unimpaired (CU). The mean MMSE score was 27.18 (SD = 3.1). Educational levels varied, with 6.3% having ≤8 years of education, 57.7% with a high school diploma, 25.3% with higher degrees, and 10.7% with a master's degree or higher. Blood draws were collected to examine AD/ADRD plasma biomarkers and common comorbidity markers. Additionally, 31.2% carried one or more APOE ε4 alleles.

**Conclusions:**

These findings demonstrate the success of our innovative MADRE initiative in recruiting and retaining B/AA participants for comprehensive in‐home evaluations, emphasizing the effectiveness of culturally relevant, accessible research methods for underserved populations.

